# Treatment selection in multi-arm, multi-stage clinical trials

**DOI:** 10.1186/1745-6215-14-S1-O4

**Published:** 2013-11-29

**Authors:** Thomas Jaki, Dominic Magirr, Nigel Stallard

**Affiliations:** 1 Lancaster University, Lancaster, UK; 2 Warwick Medical School, Warwick, UK

## 

Adaptive designs that are based on group-sequential approaches have the benefit of being efficient as stopping boundaries can be found that lead to good operating characteristics with test decisions based solely on sufficient statistics. The drawback of these so called “pre-planned adaptive” designs is that unexpected design changes are not possible without impacting the error rates. “Flexible adaptive designs”, and in particular designs based on p-value combination, on the other hand can cope with a large number of contingencies at the cost of reduced efficiency.

In this presentation we focus on so called multi-arm multi-stage trials which compare several active treatments against control at a series of interim analysis. We will discuss how these “pre-planned adaptive designs” can be modified to allow for flexibility. We then show how the added flexibility can be used for treatment selection and evaluate the impact on power in a simulation study. The results show that a combination of a well chosen pre-planned design and an application of the conditional error principle to allow flexible treatment selection results in an impressive overall procedure.

